# Supplementary figures and images for: Hydrogen peroxide induced loss of heterozygosity correlates with replicative lifespan and mitotic asymmetry in Saccharomyces cerevisiae
Source: PeerJ. 2016 Nov 3;4:e2671. doi: 10.7717/peerj.2671 (PMC5101604; doi:10.7717/peerj.2671)

## H2O2 induced cell death and LOH co-vary

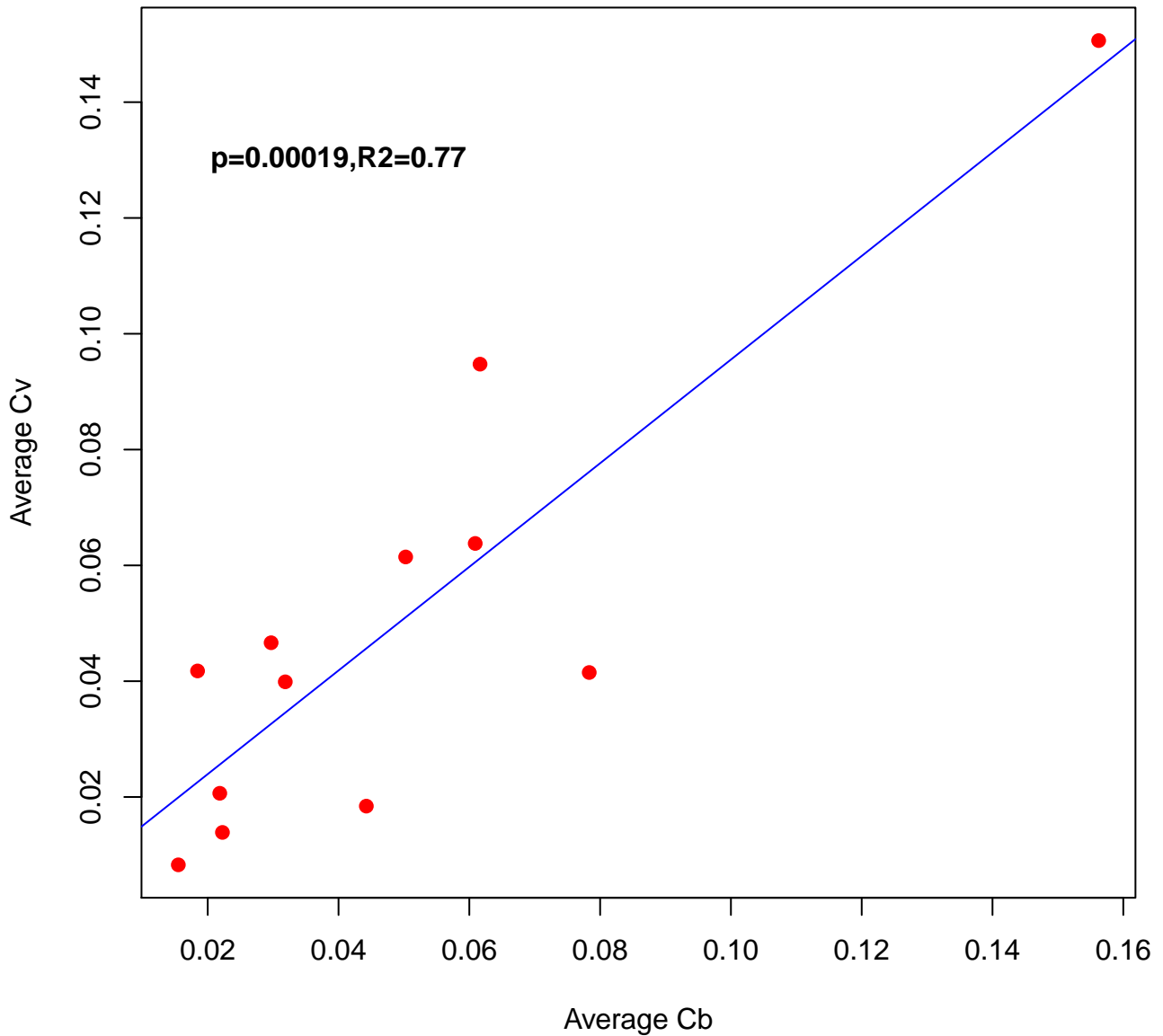

Supplement: Supplemental Information 3 [file peerj-04-2671-s003.pdf]

# BY4743 2013 May 30

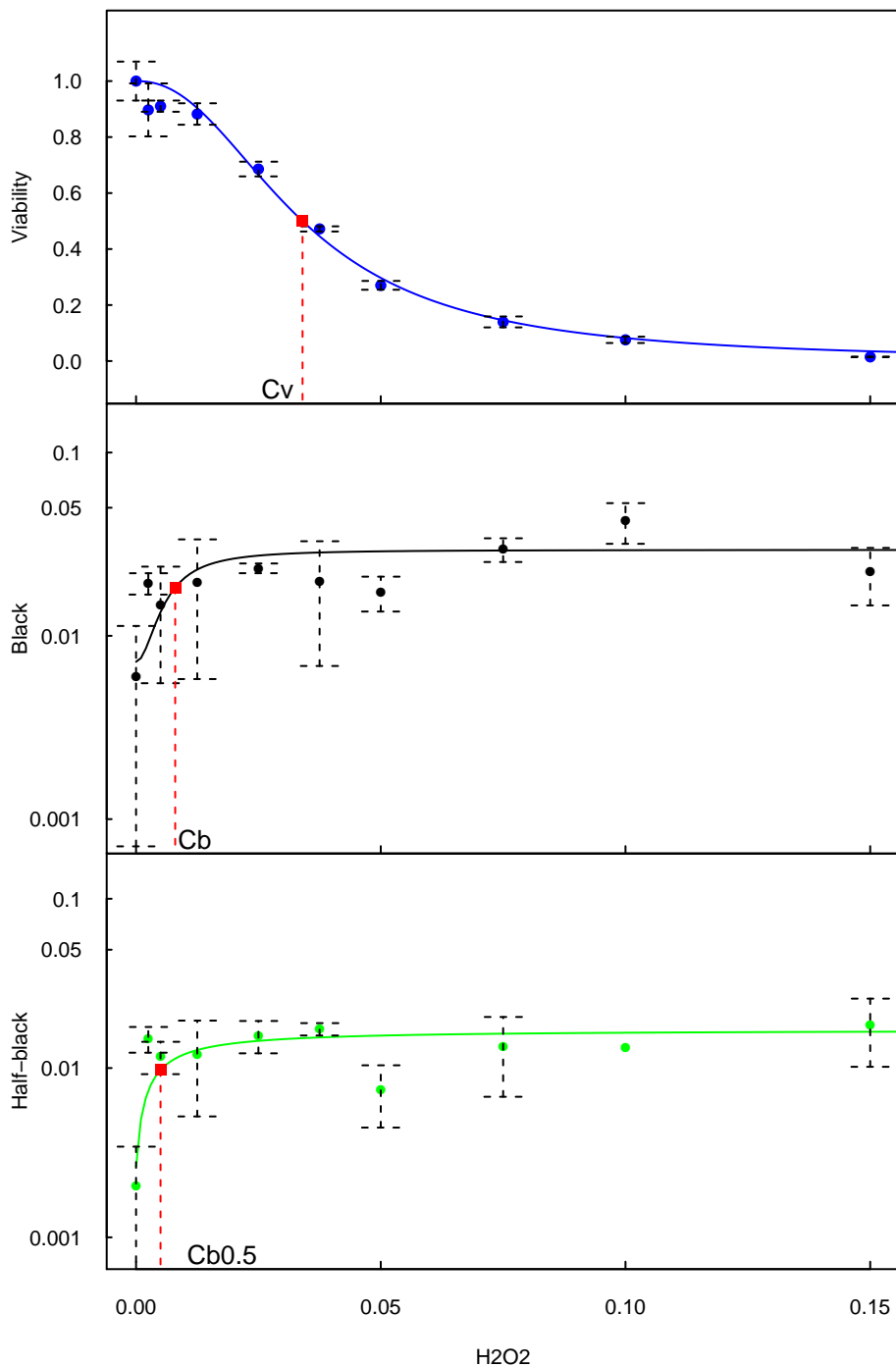

Supplement: Supplemental Information 4 — The vertical axes indicate the fractions of viabilities, black colonies, and half-black colonies. The concentrations of hydrogen peroxide in the horizontal axes are indicated in percentages estimated by volumes. Dashed bars indicate standard deviations. Red squares indicate the mid-transition points. [file peerj-04-2671-s004.pdf]

# M1-2 2011 April 22

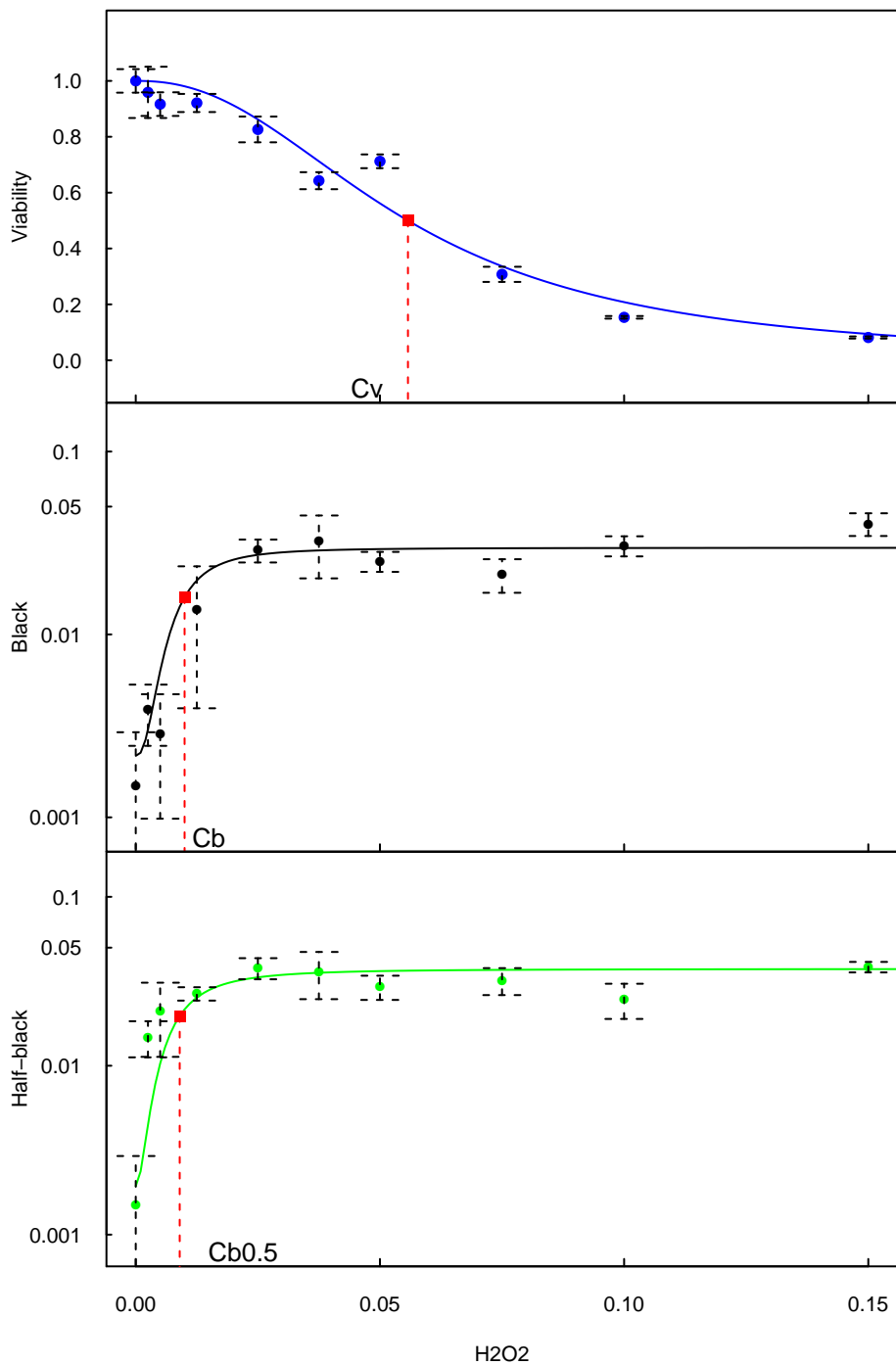

Supplement: Supplemental Information 5 — The vertical axes indicate the fractions of viabilities, black colonies, and half-black colonies. The concentrations of hydrogen peroxide in the horizontal axes are indicated in percentages estimated by volumes. Dashed bars indicate standard deviations. Red squares indicate the mid-transition points. [file peerj-04-2671-s005.pdf]

M2-8 2011 Dec 19

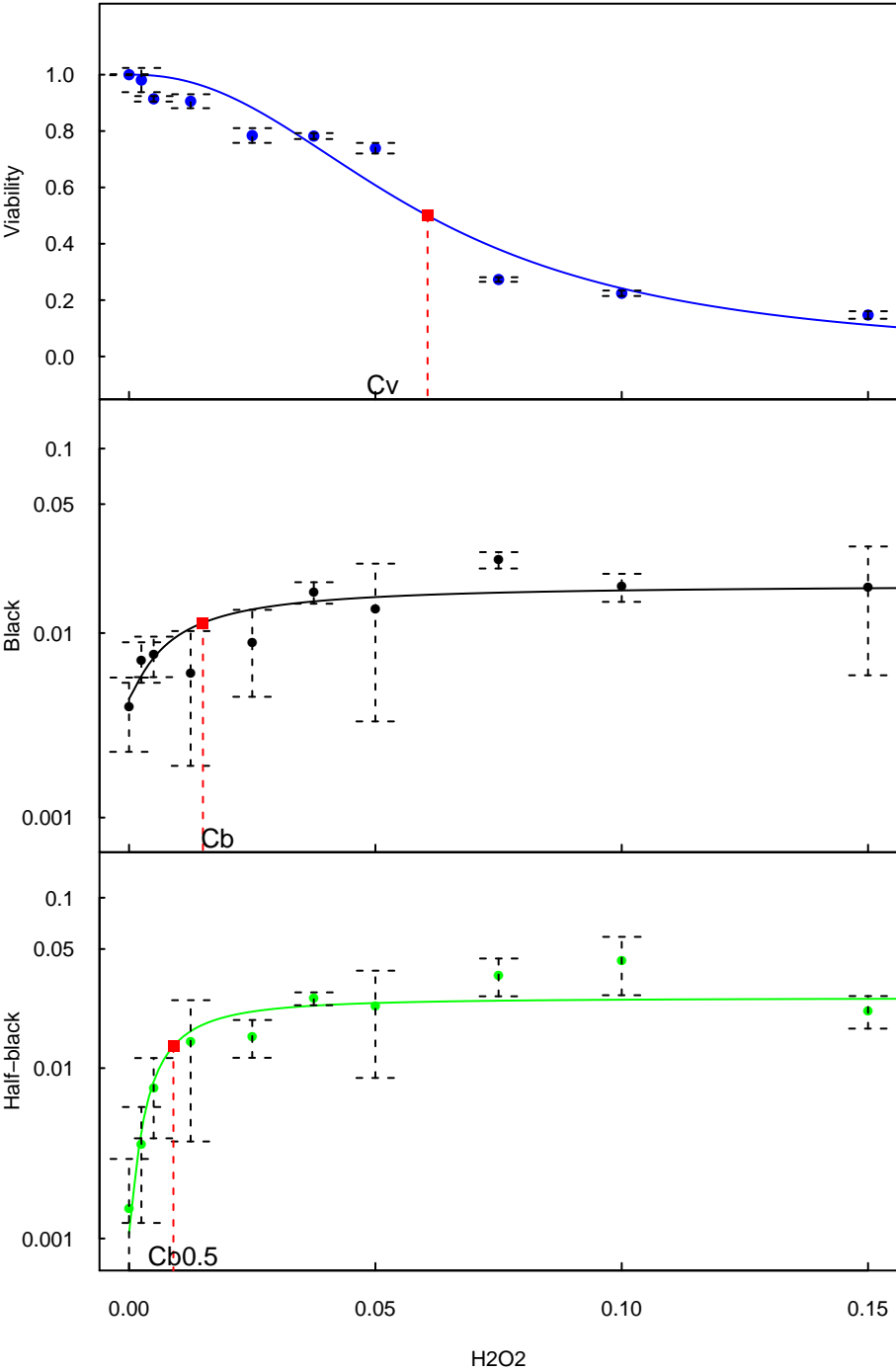

Supplement: Supplemental Information 6 — The vertical axes indicate the fractions of viabilities, black colonies, and half-black colonies. The concentrations of hydrogen peroxide in the horizontal axes are indicated in percentages estimated by volumes. Dashed bars indicate standard deviations. Red squares indicate the mid-transition points. [file peerj-04-2671-s006.pdf]

# M5 2011 August 16

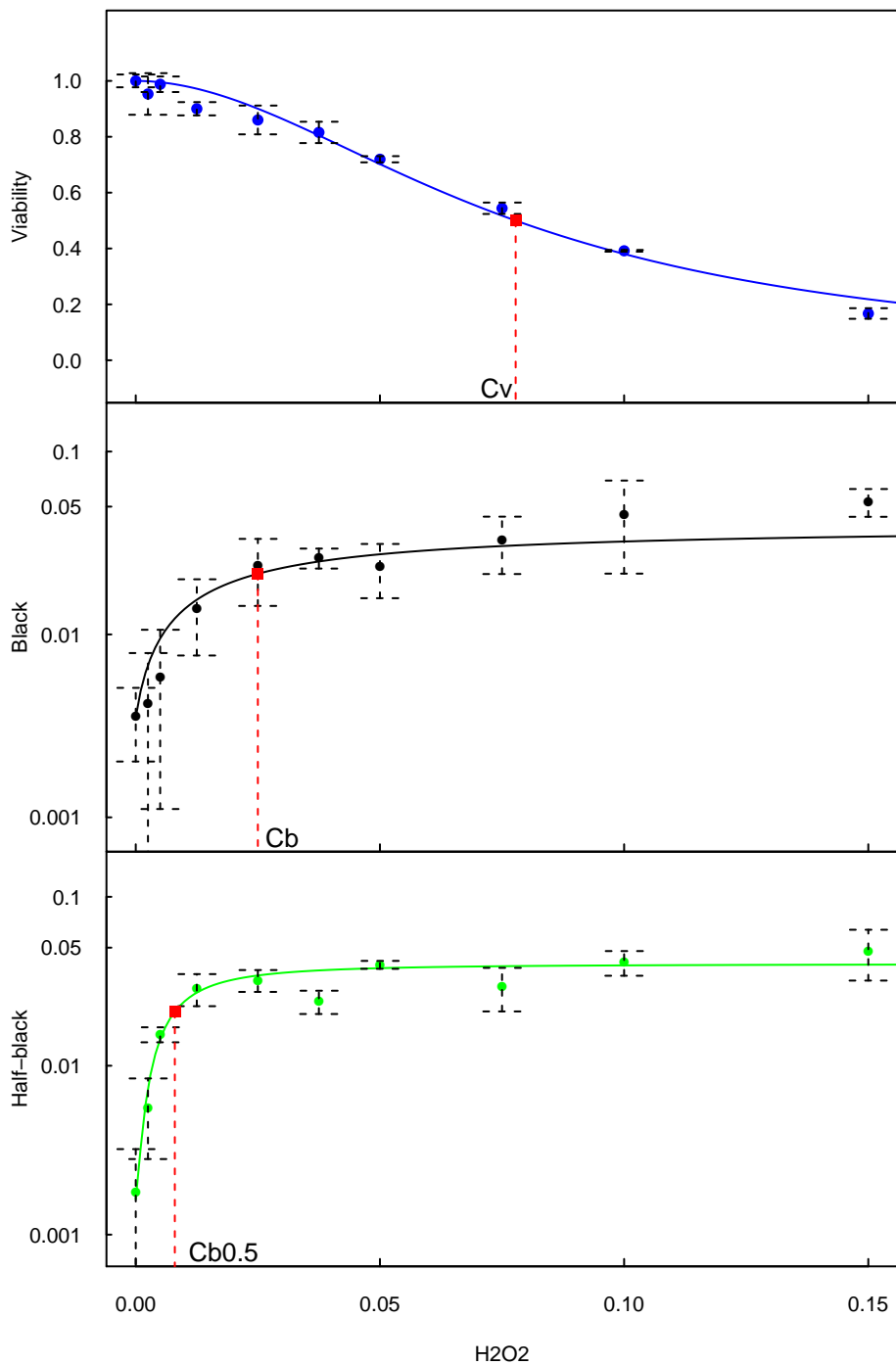

Supplement: Supplemental Information 7 — The vertical axes indicate the fractions of viabilities, black colonies, and half-black colonies. The concentrations of hydrogen peroxide in the horizontal axes are indicated in percentages estimated by volumes. Dashed bars indicate standard deviations. Red squares indicate the mid-transition points. [file peerj-04-2671-s007.pdf]

# M8 2011 Mar 30

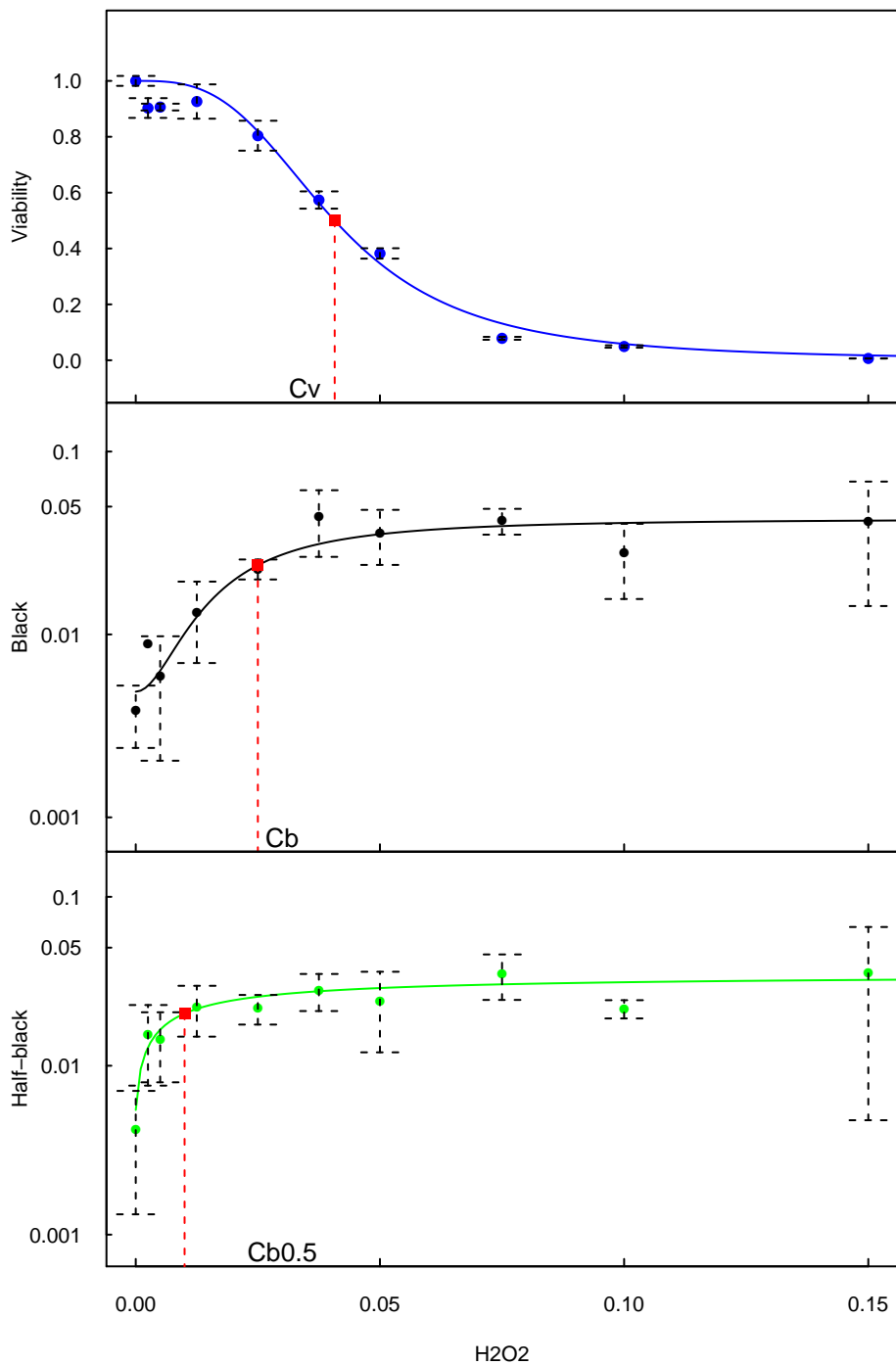

Supplement: Supplemental Information 8 — The vertical axes indicate the fractions of viabilities, black colonies, and half-black colonies. The concentrations of hydrogen peroxide in the horizontal axes are indicated in percentages estimated by volumes. Dashed bars indicate standard deviations. Red squares indicate the mid-transition points. [file peerj-04-2671-s008.pdf]

# M13 2011 May 18

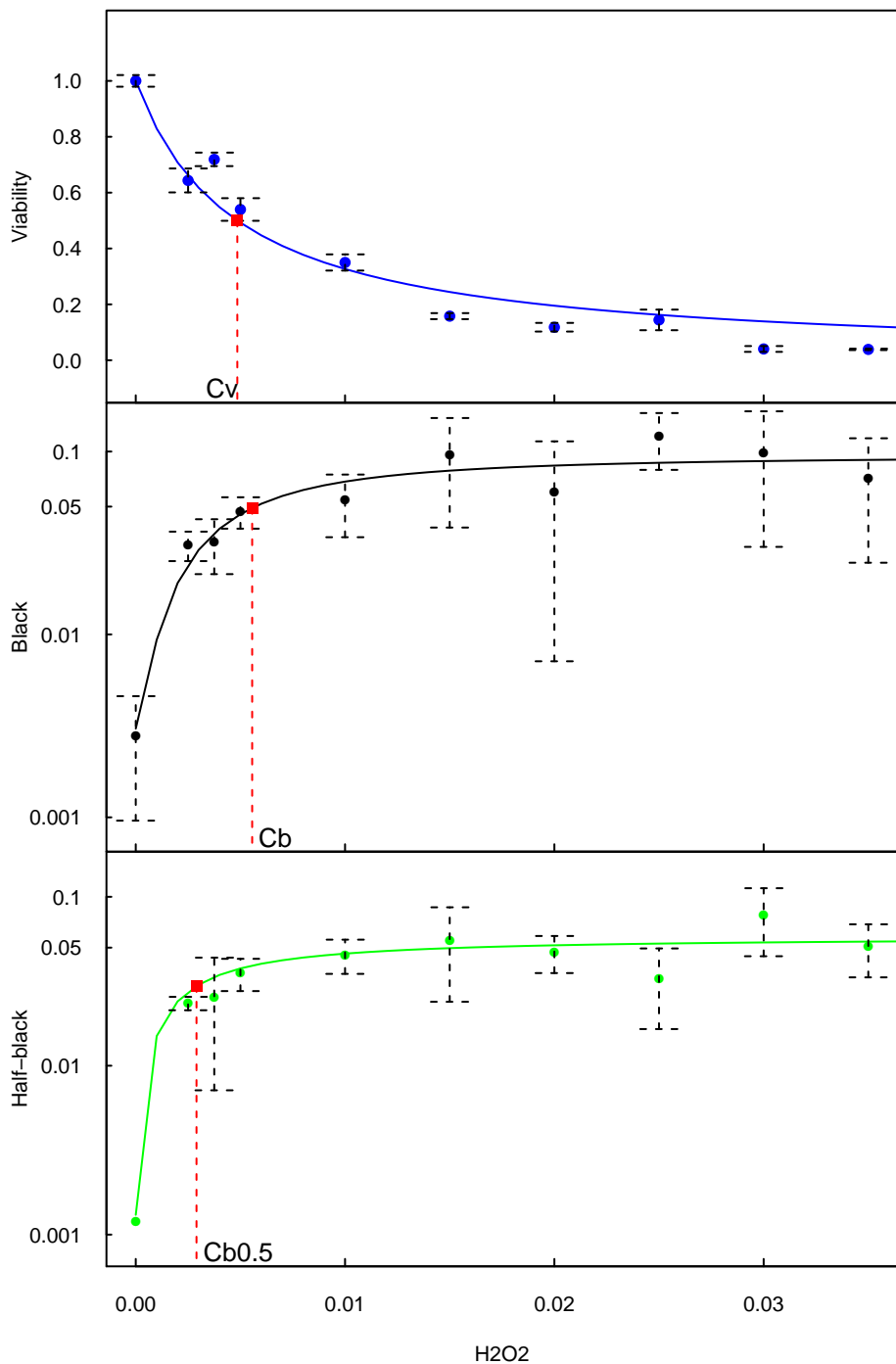

Supplement: Supplemental Information 9 — The vertical axes indicate the fractions of viabilities, black colonies, and half-black colonies. The concentrations of hydrogen peroxide in the horizontal axes are indicated in percentages estimated by volumes. Dashed bars indicate standard deviations. Red squares indicate the mid-transition points. [file peerj-04-2671-s009.pdf]

# M32 2012 January 4

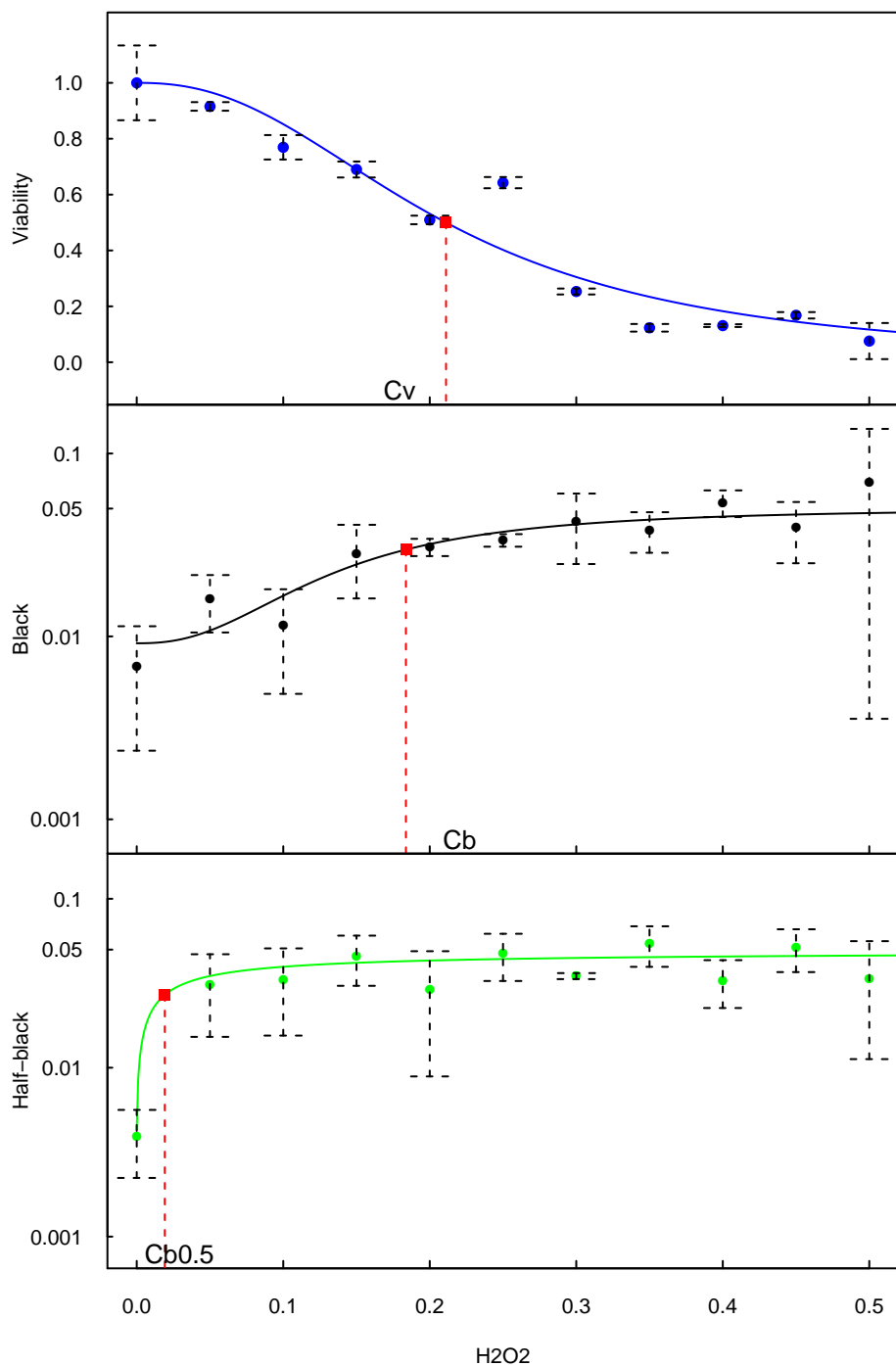

Supplement: Supplemental Information 10 — The vertical axes indicate the fractions of viabilities, black colonies, and half-black colonies. The concentrations of hydrogen peroxide in the horizontal axes are indicated in percentages estimated by volumes. Dashed bars indicate standard deviations. Red squares indicate the mid-transition points. [file peerj-04-2671-s010.pdf]

# M34 2011 August 23

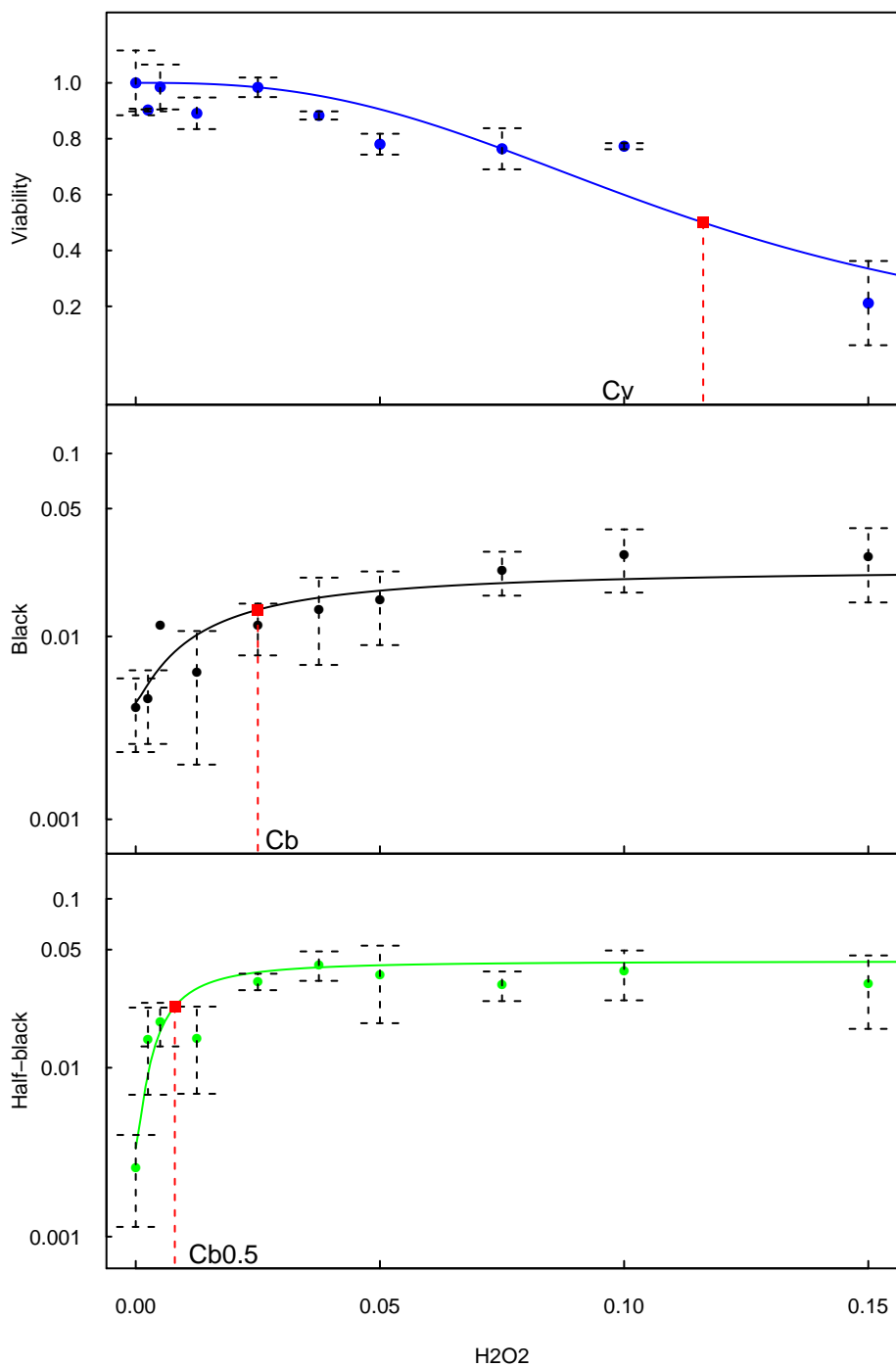

Supplement: Supplemental Information 11 — The vertical axes indicate the fractions of viabilities, black colonies, and half-black colonies. The concentrations of hydrogen peroxide in the horizontal axes are indicated in percentages estimated by volumes. Dashed bars indicate standard deviations. Red squares indicate the mid-transition points. [file peerj-04-2671-s011.pdf]

# YPS128 2011 Dec 9

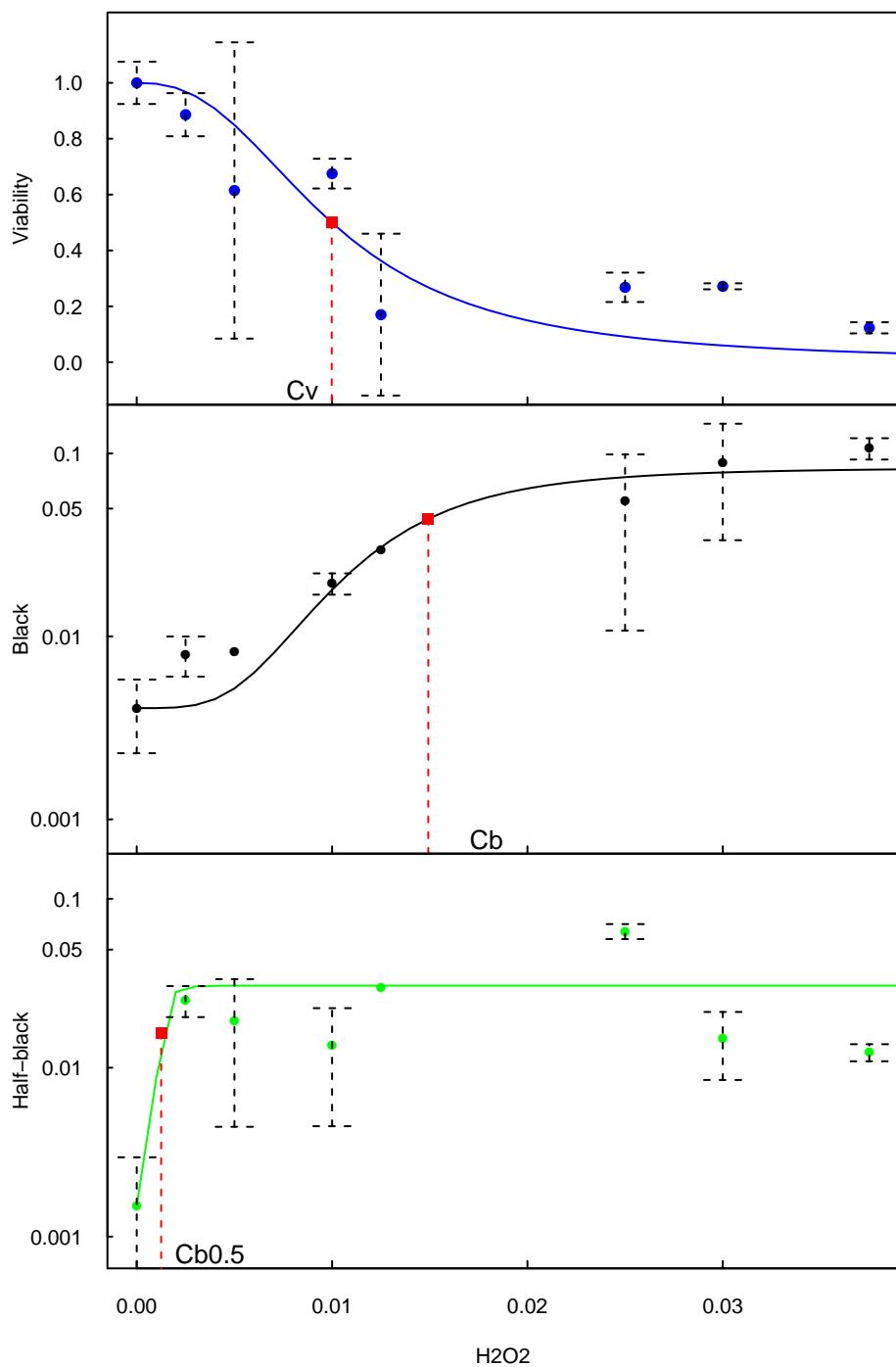

Supplement: Supplemental Information 12 — The vertical axes indicate the fractions of viabilities, black colonies, and half-black colonies. The concentrations of hydrogen peroxide in the horizontal axes are indicated in percentages estimated by volumes. Dashed bars indicate standard deviations. Red squares indicate the mid-transition points. [file peerj-04-2671-s012.pdf]

# SGU57 2012 January 12

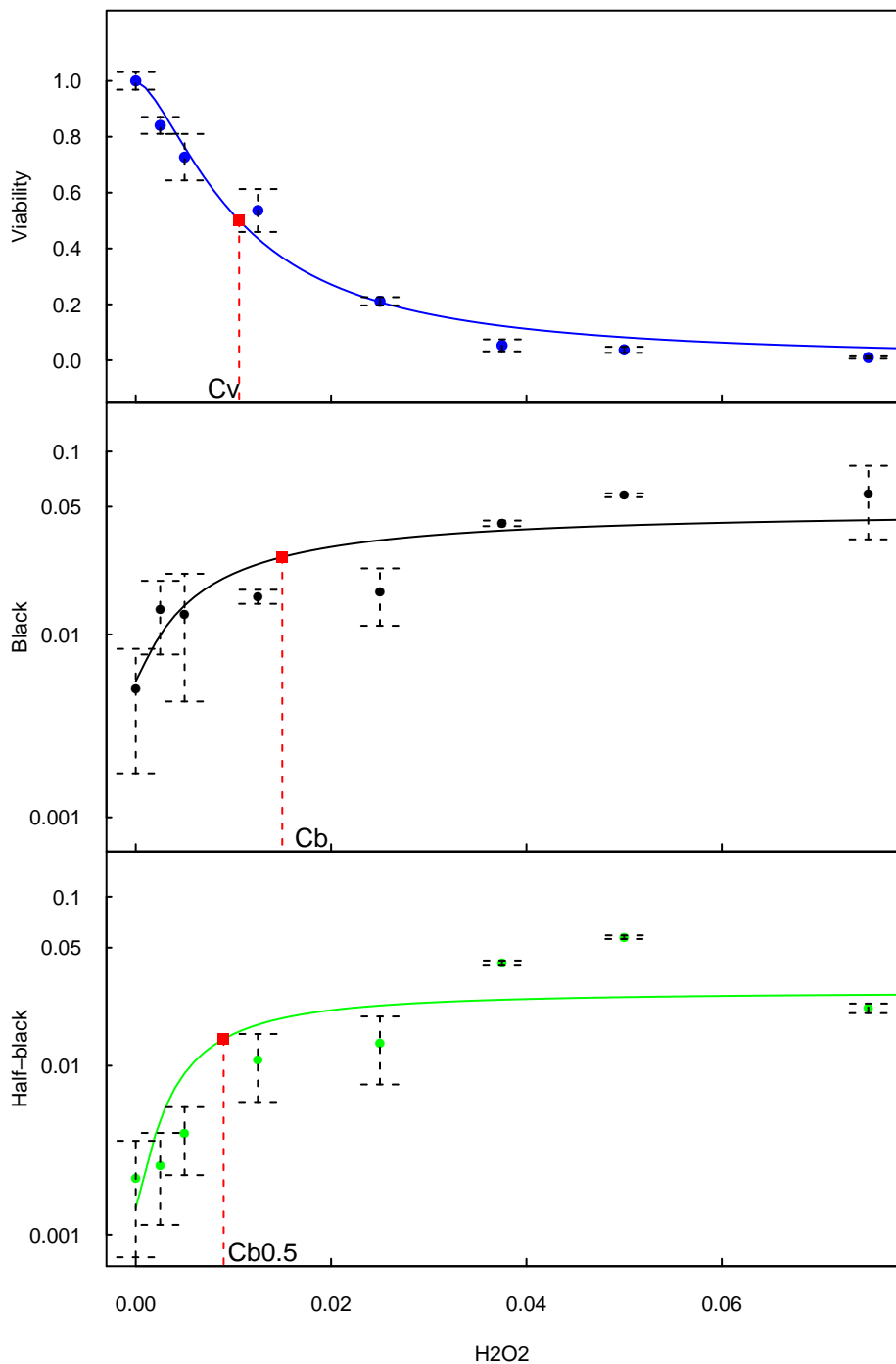

Supplement: Supplemental Information 13 — The vertical axes indicate the fractions of viabilities, black colonies, and half-black colonies. The concentrations of hydrogen peroxide in the horizontal axes are indicated in percentages estimated by volumes. Dashed bars indicate standard deviations. Red squares indicate the mid-transition points. [file peerj-04-2671-s013.pdf]
